# DEFINING DEMENTIA-FRIENDLY COMMUNITIES FROM THE PERSPECTIVE OF THOSE AFFECTED: A QUALITATIVE APPROACH

**DOI:** 10.1093/geroni/igz038.1694

**Published:** 2019-11-08

**Authors:** Iris A Wernher

**Affiliations:** Portland State University, Institute on Aging, Portland, Oregon, United States

## Abstract

Cities and communities across the globe are pledging to become more “dementia friendly,” yet many communities lack direction as to what this pledge might entail. This poster describes findings from a qualitative study conducted in and around Portland, Oregon. The goal was to better understand how communities can become more dementia friendly – from the perspective of those directly affected by the disease. The study further aimed to clarify how a city or community’s age- and dementia-friendly efforts can be integrated. Fifty community-dwelling participants – 25 individuals living with dementia and their 25 informal care partners – were interviewed separately. The questions centered on the participants’ daily lives, barriers to and opportunities for realizing desired activities, and the participants’ thoughts on how communities can become better and more inclusive places for people living with dementia. The analysis of the interviews yielded common themes, such as social inclusion, public awareness, and transportation, which served to develop a framework of dementia friendliness. This framework was compared to the World Health Organization’s framework of age friendliness to identify areas of overlap and divergence, providing the foundation for a synergistic integration of dementia-friendly initiatives into the greater context of age friendliness. Finally, the answers of individuals living with dementia and those of their care partners were compared to identify similarities and differences in their perspectives. The study was funded, in part, by Oregon citizens through the Alzheimer’s Disease Research Fund of the Oregon Charitable Checkoff Program, administered by the Oregon Partnership for Alzheimer’s Research.

